# Cannula breakage during 25G+ minimally invasive vitrectomy: a case report

**DOI:** 10.1186/s12893-020-01001-5

**Published:** 2021-03-29

**Authors:** Zhimin Shu, Siyan Jin, Chenli Shan, Linlin Ma, Jia Liu, Ning Yang, Jinsong Zhao

**Affiliations:** Department of Ophthalmology, Jilin University Second Hospital, Yatai Street, Changchun, 130000 Jilin China

**Keywords:** Cannula breakage, Sclerotomy, Vitrectomy, Minimally invasive surgery, Case report

## Abstract

**Background:**

With the continuous improvement of surgical instruments in vitrectomy, the use of a trocar and cannula not only optimizes the incision process but also facilitates insertion and withdrawal of instruments during the procedure. Nevertheless, incision-related complications have also been reported in the literature. However, cannula fractures during 25G+ minimally invasive vitrectomy have rarely been reported.

**Case presentation:**

A 62-year-old man underwent 25G+ pars plana vitrectomy for proliferative diabetic retinopathy. At the beginning of the operation, we used a trocar with a cannula to perform the sclerotomy. After the trocar was pulled out, the cannula was not seen on the surface of the sclera. Thus the inside and outside of the eye were carefully searched. The broken cannula tip was found in the ciliary body corresponding to the superonasal sclerotomy site and was subsequently removed.

**Conclusions:**

Awareness regarding the risk of intraoperative fractures of 25G+ minimally invasive ocular surgical instruments is imperative. Whenever a broken or missing cannula is encountered, the residual part should be immediately extracted to avoid revision surgeries and postoperative complications.

## Background

In 1970, the first vitrectomy machine was invented and 17-gauge (17G) pars plana vitrectomy was performed [1]. Subsequently, O'Malley introduced the 20G three-port vitrectomy system, which involved a 0.89-mm scleral incision instead of the 1.5-mm incision used in 17G vitrectomy [2]. Although the size of the scleral incision was reduced**,** incision-related complications such as hemorrhage, tissue proliferation around the incision, and peripheral retinal holes caused by pulling of the vitreous base area still occur. In 2001**,** the 25G trocar cannula system was designed. The scleral incision was much smaller, and no suture was needed. It was then that vitrectomy entered the era of minimally invasive surgery. Although the complications related to large scleral incisions considerably reduced, the softness of the instrument limited its application [3].

In 2005, Eckardt improved the 25G system to develop a 23G vitrectomy system [4], which combined the characteristics of sutureless, small-incision surgery with the inherent hardness of 20G instruments. However, intraocular surgery was much easier with 25G+ systems than with the 23G system, with better scleral incision closure rates. In 2010, Oshima introduced the 27G vitrectomy system. Although the fluid flow and cutting efficiency were lower, incision site-related complications were further reduced [5]. Despite the advances in vitrectomy instruments, there is a risk of incision site leakage in sutureless procedures, and this results in low intraocular pressure, choroidal detachment, and even endophthalmitis. In addition, there are some reports of cannula fracture at the scleral incision site that caused serious complications [6, 7]. Here we report a case of cannula fracture during 25G+ minimally invasive vitrectomy in a 62-year-old man.

## Case presentation

**Fig. 1:**
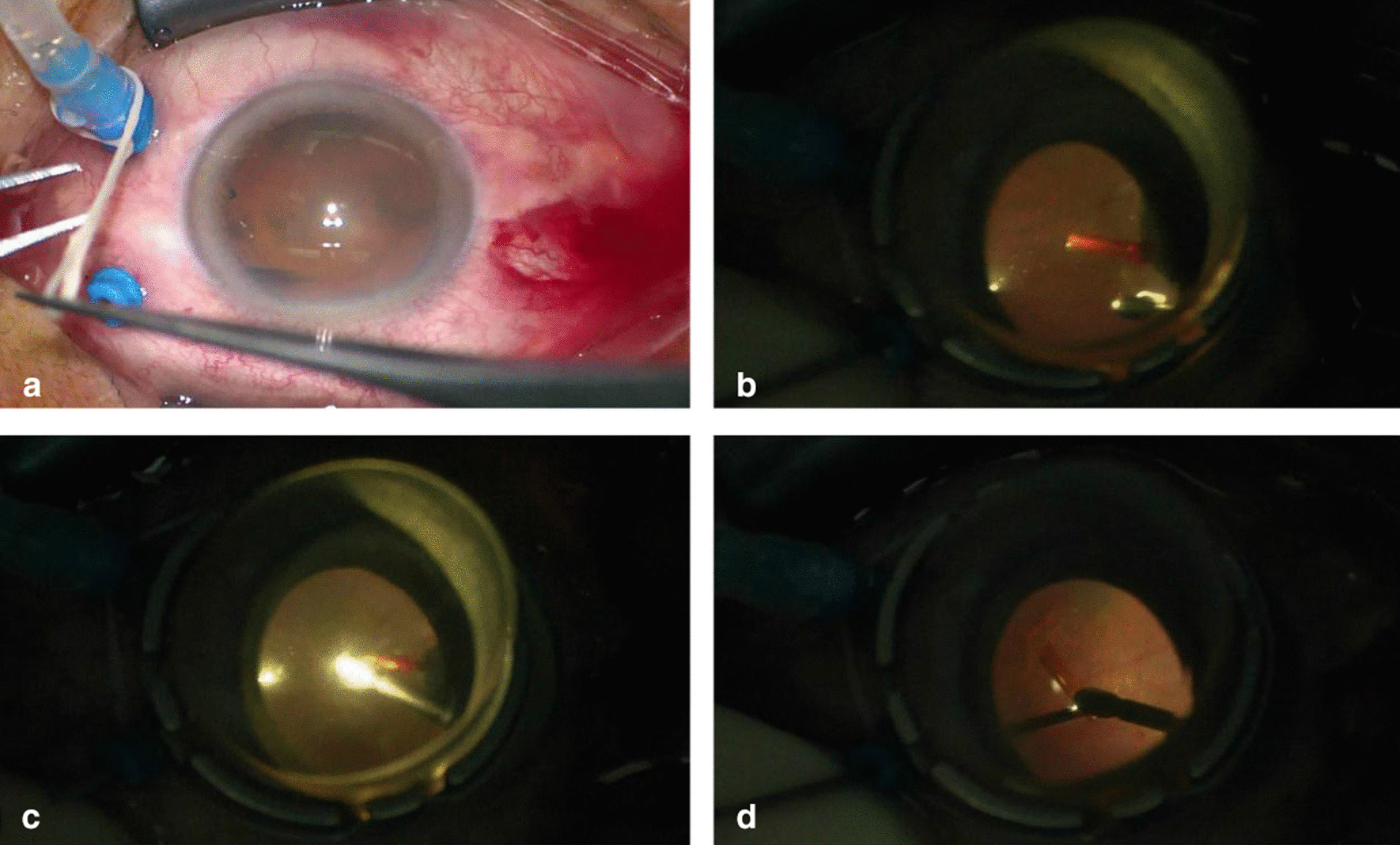
Retrieval of the broken cannula during 25-gauge pars plana vitrectomy in a 62-year-old man. **a** The trocar is extracted from the superonasal sclerotomy. **b** The broken tip of the cannula is seen in the pars plana region of the ciliary body, corresponding to the original scleral incision. **c** The foreign body forceps clamping the broken tip of the cannula. **d** With the help of the light probe, the fractured cannula is extracted

A 62-year-old man with no history of eye surgery underwent 25G+ pars plana vitrectomy for proliferative diabetic retinopathy. After retrobulbar anesthesia, a vision system was used to view the surgical field, and an inferotemporal sclerotomy was created using a 25G+ trocar. The trocar obliquely punctured the bulbar conjunctiva and sclera at an angle of 45° (sclerotomy), approximately 4 mm from the limbus and parallel to it. The trocar was twisted into the eyeball, and its orientation was gradually changed perpendicular to the limbus. After confirming that the cannula was inside the eye, we pulled out the trocar and inserted an infusion cannula into the port. Two more sclerotomies were created in the superotemporal and superonasal quadrants. However, when we pulled out the superonasal trocar, the collar of the cannula fell off and the cannula tube was missing  (Fig. 1a). Therefore, a bulbar conjunctival incision was placed to search for the missing cannula. No cannula was found in the subconjunctival space. We used a scleral depressor to look for the missing cannula tube along the peripheral retina and found the broken cannula tip in the ciliary body corresponding to the superonasal sclerotomy site. We created a new sclerotomy 2 mm above the original incision and 4 mm from the limbus and subsequently performed vitrectomy around the broken cannula to mobilize it for extraction. The new scleral incision was extended to 3 mm parallel to the limbus and allowed entry of a foreign body forceps (Fig. 1b). The broken tip of the cannula was brought into view by indenting the sclera adjacent to the original sclerotomy. We used the foreign body forceps to clamp and pull the fractured cannula toward the central vitreous body  (Fig. 1c). With the help of the light probe, the longitudinal length of the cannula was aligned with the foreign body forceps and retrieved through the enlarged incision  (Fig. 1d). No leakage occurred from the original sutureless scleral incision. The extended scleral incision was sutured with 7-0 polyglactin 910 suture.

## Discussion and conclusion

Several cases of cannula fracture have been reported. In a 72-year-old woman who underwent surgery for epiretinal membrane, half of the cannula tip was missing at the end of the vitrectomy. The following day, there was severe choroidal detachment associated with hypotony. A second surgery was performed to drain the subchoroidal hemorrhage and fluid and remove the tip of the cannula stuck inside the sclera [6]. Wong et al. described a female patient with endophthalmitis following vitrectomy. A second vitrectomy was performed, during which the surgeon identified a small subconjunctival abscess located in the inferotemporal quadrant, 3–4 mm from the limbus. After abscess drainage, a yellow plastic tubular fragment was found retained within the transscleral space. They completed the vitrectomy and removed the yellow plastic tube at the end of the procedure. The tubular fragment resembled a broken small-gauge vitrectomy cannula and measured 2 mm in length and 0.5 mm in diameter. The tip of the cannula was still missing and was not detected by the surgeon [7]. In such instances, the tip of the cannula can act as a bridge between the vitreous cavity and the suprachoroidal or subconjunctival space, resulting in postoperative complications. When vitreous fluid along the broken end of the cannula enters into the suprachoroidal space, it causes choroidal detachment and hypotony. Bleeding from the scleral wound with hypotony might have resulted in the suprachoroidal hemorrhage. Bacteria on the ocular surface may enter the vitreous cavity along the residual cannula from unsutured conjunctival and scleral puncture port resulting in endophthalmitis.

Several surgical instruments such as vitreous cutters, tano forceps, and trocars have fractured during surgical procedures. Among them, Makoto et al. found that a 25G vitreous cutter was broken during vitreous cutting [8]. Karl et al. reported a 20G tano forceps breakage during vitrectomy [9]. When Joshua et al. used a 23G trocar for puncture, the tip was adsent when the trocar was withdrawn [10]. In the above studies, fractures of different gauge and types of surgical instruments during the operation were reported. Since surgical instrument fractures are rare, no literature specifically reports their incidence. There is no report of the breakage of 20G or 23G cannula during surgery. The broken instruments reported by Chen [6] and Robert [7] were 25G, the same as in our incident. When compared with 20G and 23G instruments, 25G+ surgical instruments are more slender and fragile. The cannula, which is made of plastic and polyamide tubing, is not as strong as stainless steel instruments, which further increases the risk of fracture. In our case, we used a brand new, disposable 25G+ trocar with a 25G+ cannula, thus eliminating the possibility of device incompatibility and wear and tear.

We speculate that the cannula fracture may be related to the trocar slipping inside the cannula during scleral puncture. The cannula is locked in the trocar during scleral puncture. When the direction of the trocar entering the sclera changes from parallel to perpendicular relative to the limbus, there might be slippage between the cannula and trocar. However, the pars plana and vitreous base at the corresponding incision site may help in stabilizing the cannula tip. When the direction of trocar removal is not similar to that of the cannula, the force acting on the cannula may break it. This explains why the collar of the cannula detaches from the scleral surface while the tip of cannula remains in the eye.

Awareness regarding the risk of intraoperative fracture of 25G+ minimally invasive ocular surgical instruments is imperative. First, we should confirm the integrity of the trocar and cannula before puncturing. Then the direction of the force during puncturing must correspond to the long axis of the cannula. When resistance is encountered, violent puncture must be avoided. We can select a suitable puncture port to perform repeat puncturing. To aid with early detection and retrieval, we should always confirm the integrity of the cannula after removal of the trocar and at the end of the surgery, after pulling out the cannula. Whenever a broken or missing cannula is encountered, the residual part should be immediately extracted to avoid revision surgeries and postoperative complications. In addition, the manufacturer has improved the material and selected stronger and better mechanical materials, which can further reduce the occurrence of cannula breakage during the operation.

## Data Availability

All data analyzed was included in this published case report.
